# Evaluating the Psychometric Properties and Clinical Utility of a Digital Psychosocial Self-Screening Tool (HEARTSMAP-U) for Postsecondary Students: Prospective Cohort Study

**DOI:** 10.2196/48709

**Published:** 2023-08-09

**Authors:** Punit Virk, Ravia Arora, Heather Burt, Caitlin Finnamore, Anne Gadermann, Skye Barbic, Quynh Doan

**Affiliations:** 1 School of Population and Public Health The University of British Columbia Vancouver, BC Canada; 2 BC Children's Hospital Research Institute Vancouver, BC Canada; 3 Cumming School of Medicine University of Calgary Calgary, AB Canada; 4 Centre for Health Evaluation and Outcome Sciences Providence Health Vancouver, BC Canada; 5 Department of Occupational Science and Occupational Therapy Faculty of Medicine The University of British Columbia Vancouver, BC Canada; 6 Department of Paediatrics Faculty of Medicine The University of British Columbia Vancouver, BC Canada

**Keywords:** mental health, screening, validity, postsecondary students, clinical utility

## Abstract

**Background:**

Existing screening tools for mental health issues among postsecondary students have several challenges, including a lack of standardization and codevelopment by students. HEARTSMAP-U was adapted to address these issues.

**Objective:**

This study aimed to evaluate the suitability of HEARTSMAP-U as a self-screening tool for psychosocial issues among postsecondary students by evaluating its validity evidence and clinical utility.

**Methods:**

A prospective cohort study was conducted with University of British Columbia Vancouver students to evaluate HEARTSMAP-U’s predictive validity and convergent validity. Participating students completed baseline and 3-month follow-up assessments via HEARTSMAP-U and a clinician-administered interview.

**Results:**

In a diverse student sample (n=100), HEARTSMAP-U demonstrated high sensitivity (95%-100%) in identifying any psychiatric concerns that were flagged by a research clinician, with lower specificity (21%-25%). Strong convergent validity (*r*=0.54-0.68) was demonstrated when relevant domains and sections of HEARTSMAP-U were compared with those of other conceptually similar instruments.

**Conclusions:**

This preliminary evaluation suggests that HEARTSMAP-U may be suitable for screening in the postsecondary educational setting. However, a larger-scale evaluation is necessary to confirm and expand on these findings.

## Introduction

### Background

The prevalence of diagnosable mental health challenges and generalized psychological distress is rising in the postsecondary student population. In an American student sample from 196 postsecondary institutions (n=155,026), lifetime mental health diagnoses increased from 22% (2007) to 36% (2017), and the percentage of treatment seeking increased from 19% (2007) to 34% (2017) [1]. Similarly, in the Canadian context, between 2013 (n=22,995) and 2019 (n=38,127), the proportion of students who self-reported a diagnosis of anxiety, depression, an addiction, an eating disorder, bipolar disorder, and schizophrenia from a medical provider increased [2]. Past-year diagnosis of any mental health disorder and formal treatment seeking rose from 22% (2013) to 37% (2019) and 31% (2013) to 37% (2019), respectively [2]. Unfortunately, poor access to care, long wait times due to service saturation, complex referral requirements, and uncoordinated care pathways have been longstanding barriers for students accessing care within postsecondary educational environments [3-6].

The university context is an important venue to consider for detecting untreated mental health issues. In fact, Canada’s *National Standard for Post-Secondary Student Mental Health and Well-being* calls for postsecondary institutions to assess opportunities for early detection within their health systems [7]. Universal screening and resource navigational interventions can provide institutions with the necessary infrastructure to promote early detection and intervention of psychological (eg, depression and anxiety) and social challenges (eg, relationships and housing), which we hereafter refer to as psychosocial challenge [8-11]. Psychosocial health is considered a multifaceted term denoting the mental, emotional, and social dimensions that shape and make up individuals’ well-being [12]. Moreover, screening tools may support students in reflecting on their mental health status as well as building self-awareness and mental health literacy [13,14]. This is especially important given the fact that low perceived need is a common help-seeking barrier [5]. In addition, universal screening is an approach by which all students may have an equal opportunity for early identification and service acquisition [15] and may offer an effective strategy for reaching equity-seeking groups (eg, racialized, disabled, gender, and sexual minority groups) [8,16-18]. In a campus-wide universal screening campaign open to all students, Kodish et al [16] observed substantial participation of a racially diverse student population (73.3%). Although racialized students were less likely to have received prior mental health treatment compared with non-Hispanic White students, they were just as likely to initiate treatment after completing screening. However, the implementation and integration of screening tools within campus health systems remains variable across institutions [19].

Several challenges that hinder effective screening integration in postsecondary health systems persist. First, at the health system level, positive screens have serious implications for system capacity and adequate service provision. Second, most instruments lack codevelopment with students, neglecting to reflect what students consider important to their psychological and social well-being [8,17,20,21]. Among studies evaluating student-specific psychosocial screening instruments, there is insufficient reporting on how these tools reflect what students perceive to be important to their psychosocial health and well-being [8,17,20,21]. For example, the Counseling Center Assessment of Psychological Symptoms 62-item scale was developed to facilitate multidomain psychosocial self-assessment among postsecondary students across 8 domains: depression, generalized anxiety, social anxiety, academic distress, eating concerns, frustration or anger, alcohol use, and distress [22]. However, there is a paucity of published literature reporting on the tool’s content validity, specifically on students’ perception of the tool’s relevance to their lived experiences and the perceived acceptability of its psychosocial coverage. To address the need for a student-centered, multidomain psychosocial screening tool, we adapted existing pediatric, clinician-administered (HEARTSMAP) and patient-administered (MyHEARTSMAP) instruments into HEARTSMAP-U, a version suitable for postsecondary students. We adopted a student-centered approach, which addressed the aforementioned challenges with existing tools and helped us ensure that HEARTSMAP-U’s content was relevant, applicable, and acceptable to students [23].

Third, many existing instruments are based on diagnostic criteria and focus on specific mental health conditions (eg, depression and anxiety) [24,25]. However, multidomain screening tools, such as HEARTSMAP-U, allow for a broader and more holistic assessment of psychosocial stressors experienced by students and may identify nonspecific and subthreshold issues that may not get captured by stringent diagnostic criteria [26].

Fourth, most available instruments consist exclusively of a screening component, often evaluating symptoms or functional impairments on a Likert-style scale. However, systematic reviews and meta-analyses have found that screening alone may have limited impact on patients’ health outcomes and health-related quality of life (HRQOL) [24,25]. A critical issue with only screening is the need for adequate service provisions for positive screens. Therefore, the US Preventative Services Task Force recommends routine depression screening only when adequate treatment and follow-up systems are in place [27]. Without these provisions or resource information, students may feel ill-equipped to navigate through campus and community-based mental health services [11]. However, the help-seeking impact of combining psychosocial screening with supportive interventions (eg, personalized feedback, psychoeducation, and resource navigation) remains a largely understudied area. Working within the structure of existing HEARTSMAP instruments allowed us to address these challenges by adapting an instrument with both assessment and resource navigational components. A final challenge with existing instruments is the variable degree of validity evidence supporting their measurement properties for use among postsecondary students.

### This Study

We iteratively adapted HEARTSMAP-U through extensive and ongoing engagement with postsecondary students and clinical experts. Campus-based mental health professionals played a vital role in ensuring that HEARTSMAP-U effectively captured a diverse range of clinically relevant psychosocial stressors, varying in severity, and that its content adequately addressed critical safety concerns, such as suicidality, homicidality, and abuse. The student consultation process played a crucial role in establishing a “common language” that facilitated mutual understanding of the tool’s content between student users and researchers. This involved implementing helpful features such as hover overs for technical terms, eliminating jargon, and ensuring clarity. In addition, students provided valuable feedback to ensure that the scoring descriptors were realistic and easily distinguishable [23].

Before implementing a screening instrument in the postsecondary educational setting, evidence demonstrating the instrument’s fitness for purpose as a self-screening tool for postsecondary students is needed. In the patient-reported outcome measure context, fitness for purpose refers to how suited an instrument is for a particular setting and depends on the demonstration of validity and reliability evidence and usefulness or “clinical utility” in its context of use [28,29]. Multifaceted evidence of HEARTSMAP-U’s validity and clinical utility remains to be established.

Predictive validity assesses how well an instrument can predict gold-standard performance. Clinical evaluation is the gold standard for psychosocial assessment but can be time-consuming and requires specialized training. Demonstrating HEARTSMAP-U’s predictive validity is important for determining its suitability as a screener. Convergent validity measures the correlation between scores on instruments measuring similar constructs [30]. By demonstrating HEARTSMAP-U’s convergence with similar instruments, we can assess how accurately it measures what it is intended to measure. Clinical utility refers to how well an instrument facilitates treatment planning, clinician-patient interactions, and collaboration [31]. To assess HEARTSMAP-U’s clinical utility, we examined students’ short-term help-seeking experiences and access to recommended care after self-screening.

This study aimed to determine whether HEARTSMAP-U is fit for purpose as a psychosocial self-screening tool for students in the postsecondary educational setting. The primary objective of this study was to estimate the preliminary evidence of HEARTSMAP-U’s convergent and predictive validity. The secondary objective was to estimate HEARTSMAP-U’s clinical utility or the relationship between psychosocial screening and students’ use of psychosocial resources at 3-month follow-up.

## Methods

### Design

**Figure 1:**
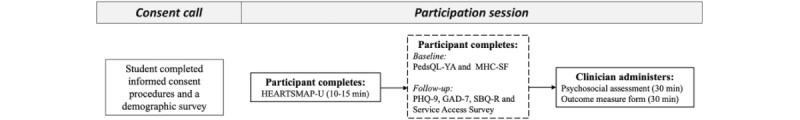
Study procedures chronologically outlined from informed consent to study participation. GAD-7: 7-item Generalized Anxiety Disorder; MHC-SF: Mental Health Continuum-Short Form; PedsQL-YA: Pediatric Quality of Life-Young Adult; PHQ-9: 9-item Patient Health Questionnaire; SBQ-R: Suicide Behaviors Questionnaire–Revised.

We conducted a prospective cohort study with 2 time points, baseline and 3-month follow-up. Baseline and follow-up sessions took place between December 2020 and April 2021 and April 2021 and July 2021, respectively. Students provided informed e-consent before participation. All study procedures were conducted remotely and individually with each participant over the Zoom videoconferencing platform (Zoom Video Communications, Inc), which involved a combination of self-report surveys and a clinician-administered interview. Participants received a CAD $60.00 (US $45.40) gift card or check upon successful completion of the baseline and follow-up sessions. All the procedures are illustrated in Figure 1.

### Ethics Approval

Approval was obtained from the University of British Columbia (UBC) Behavioural Research Ethics Board before data collection (H20-02556).

### Participants

All students aged ≥17 years enrolled at UBC Vancouver at the time of recruitment were eligible to participate. We excluded students who did not have a laptop or desktop computing device to complete study participation, were unavailable for a 3-month follow-up session, or were physically residing outside British Columbia at the time of study participation. Recruitment efforts were entirely web-based (eg, social media, e-newsletters, and institutional listserves). Study promotion efforts were supported by a student-led mental health advocacy organization.

Sampling was conducted in 2 stages. In stage 1, a consecutive, convenience-based sample of 25 eligible students was included. In stage 2, quota-based purposive sampling was conducted to ensure proportional representation and diversity across several demographic variables: gender identity, racial identity, student type (undergraduate, graduate, or professional), and self-rated mental health status (excellent, very good, good, fair, or poor). All quota proportions (Multimedia Appendix 1) were based on a combination of existing UBC institutional demographic data [32], Canadian census data [33], and the broader epidemiological literature on postsecondary students [34]. To facilitate quota-based sampling, all prospective participants completed a brief 1-minute web-based expression of interest form. Select students were invited into the study on a rolling basis.

### Measures

#### HEARTSMAP-U

HEARTSMAP-U is a self-assessment tool designed for postsecondary students to evaluate their psychosocial situation across 10 sections, including *Housing & Material Security*; *Education & Activities*; *Relationships*; *Thoughts & Anxiety*; *Substances & Behavioural Dependencies*; *Safety*; *Sexual Wellness*; *Mood*; *Abuse*; and *Professionals & Resources* [23]. HEARTSMAP-U was adapted for postsecondary students based on previously validated clinician- and patient-administered pediatric versions, HEARTSMAP and MyHEARTSMAP, respectively. The adaptation process has been thoroughly documented elsewhere [23]. Key modifications focused on ensuring that the tool’s content and language were age and developmentally appropriate to the postsecondary context of use. We worked with students and clinicians to ensure that the tool’s content was relevant (ie, student specific), comprehensible, and clinically meaningful (ie, captures a broad spectrum of concern severity). For example, the *Home* section on HEARTSMAP or MyHEARTSMAP was adapted to *Housing & Material Security*, recognizing that postsecondary students may not attribute their housing situation as what is socially understood as a “home” and that financial security and self-management is critical for this population.

Each psychosocial section consists of a single item with a 4-point Likert-type scale ranging from 0 (no concern) to 3 (severe concern) to assess for challenges specific to the psychosocial area in question. Brief descriptors accompany each scoring option to help users select the score most appropriate for their situation. In addition, in each section, if the user reports any concerns (score 1-3), they are prompted to indicate whether they have already accessed resources to address those specific concerns (yes or no). Each tool section also has an open-ended textbox that allows students to qualitatively describe their situation as it pertains to their scoring.

After users score all 10 sections, the tool’s decision-making algorithm sorts and aggregates sectional scores into broad domains (*Social*, *Functional*, *Student Health*, and *Psychiatry*), each characterized by a unique combination of services and resources, including social services, resources to support daily functioning, frontline resources (for nonpsychiatric, psychosocial needs), psychiatric resources, and services. Each domain maps to 3 sections; multiple sections map to >1 domain. The social domain consists of the *Housing & Material Security*, *Substances & Behavioural Dependencies*, and *Abuse* sections. The function domain includes the *Substances & Behavioural Dependencies*, *Relationships*, and *Sexual Wellness* sections. The psychiatry domain is composed of the *Mood*, *Thoughts & Anxiety*, and *Safety* sections. The domain score (0-9) is the composite or sum of each mapping section’s concern severity score (0-3). Each domain score is categorized based on severity, which determines the intensity of tool-generated support recommendations. Score cutoffs are based on clinical judgment and extensive validation in community and clinical samples of youths and adolescents: “none” (0), “mild” (1-3), “moderate” (4-6), or “severe” (7-9) concerns [35-41].

On the basis of students’ concern severity and urgency, HEARTSMAP-U may recommend a range of psychiatric (eg, crisis response, psychiatric consultation, counseling services, peer support services, and self-directed resources) and broader psychosocial services (eg, academic counseling, financial advising, housing services, primary care, and peer support). Recommendations may be firm (ie, urgent and time sensitive) or soft (ie, considerations and less urgent) depending on concern severity and acuity. Currently, HEARTSMAP-U does not offer direct referral to any campus- or community-based services but offers students contact and service information (eg, cost, accessibility, and hours of operation) to facilitate help seeking. A paper version of the HEARTSMAP-U instrument is provided in Multimedia Appendix 2.

#### Clinician-Administered Psychosocial Interview

The clinician-administered psychosocial interview served as a practical, real-world criterion standard against which we measured the evidence of HEARTSMAP-U’s predictive validity. Research clinicians were asked to mimic procedures (eg, questioning, probes, and rapport) they typically used when performing intake-style assessments. Although the interview was intended to be open ended and flexible to best reflect real-world assessment content, several parameters were outlined to support the clinicians. First, the interview was not intended to be diagnostic; rather, clinicians were asked to identify broad concern areas in which students demonstrated support needs, ensuring that symptoms that were not clearly defined or were subclinical were not missed. Second, clinicians were provided with a standardized data collection tool to document the various psychological and social (psychosocial) aspects shaping students’ mental health. The form was developed with 6 research clinicians to ensure a mutual understanding of reporting expectations.

Before participant recruitment, all research clinicians participated in a web-based 2-hour training session. After reviewing all the study procedures, clinicians reviewed and modified the preliminary form version developed by the study team. Clinicians proposed modifications to minimize potential reporting bias and ensure a consistent understanding and application of the form across all clinicians. To this effect, clinicians engaged in collaborative discussions to identify opportunities to improve content accuracy, clarity, and distinctions between severity levels. At the end of the session, clinicians independently applied the updated data collection form to 2 fictional vignettes, each describing students with differing mental health presentations (eg, concern type and severity). Across both cases, all clinicians consistently classified the presence or absence of concerns, concern severity, and resource needs.

The finalized version of the form consisted of 2 components: the first focused on psychiatric concerns, and the second focused on broader psychosocial issues. This was intended to ensure that in their interviews, clinicians probed and documented both social and psychological challenges. In the first component, the form assesses the presence of psychiatric concerns (yes or no), which have been operationalized as thought disturbances, anxiety, mood-related issues, and suicidal behavior. If psychiatric concerns were endorsed, the clinician was asked to holistically rate and describe concern severity: mild, moderate, or severe. At each severity level, the data collection tool had general descriptors characterizing severity-specific distress, functional impairment, and resource needs to support consistent score interpretation across participating clinicians. The form included relevant International Classification of Diseases, 10th revision coding categories for clinicians to broadly check off areas of concern that might apply to students. These codes were intended to flag suspected concern areas and were not meant to be diagnostically applied. Clinicians also had the option to “write-in” concerns that were not adequately reflected on the form. Finally, clinicians identified any outstanding psychiatric resource needs using a prespecified list of mental health resources that range in intensity (self-directed to urgent professional care). Again, clinicians had the option of writing in resources or services not mentioned on the form. For each recommended service, clinicians were asked to indicate the time frame within which the student should access the resource: immediately, within 72 hours, within 1 week, or after 1 week.

The structure and content of the second component were similar to those of the first component. Clinicians were asked to rate the presence, severity, and types of broader psychosocial issues that the student may be experiencing, as well as specific resource needs. Broader psychosocial concerns were operationalized as challenges involving the interaction of both social and psychological stressors in relation to students’ functioning (eg, relationships), environment (eg, housing and finances), behaviors (high-risk sexual activities and substance use), and development (eg, learning disabilities and cognition).

#### Pediatric Quality of Life-Young Adult Version

The Pediatric Quality of Life-Young Adult (PedsQL-YA) is a 13-item HRQOL instrument designed to measure physical, emotional, social, and work or school functioning among individuals aged 18 to 25 years. Among college students living with chronic health conditions and those living without chronic health conditions (n=1264), the PedsQL-YA has demonstrated strong internal consistency, known-group discriminant validity, and convergent validity with the Short Form-8 Health Survey [42]. For the current data, the Cronbach α coefficient for the total questionnaire was .92. For the subscales, α estimates were .81 (physical), .83 (emotional), .80 (social), and .77 (school).

#### Mental Health Continuum-Short Form

The Mental Health Continuum-Short Form (MHC-SF) is a 14-item measure of positive mental health based on a 3-factor model of well-being: emotional, psychological, and social. In cross-national samples, including Canadian people, the MHC-SF has demonstrated strong evidence of internal consistency; structural validity; and criterion validity against concepts of psychological distress and negative social interactions and the World Health Organization Disability Assessment Schedule [43-45]. In this study’s sample, the estimated Cronbach α coefficients for the subscales were .88 (emotional), .83 (psychological), and .82 (social).

#### 9-Item Patient Health Questionnaire

The 9-item Patient Health Questionnaire (PHQ-9) is a self-administered unidimensional instrument for screening Diagnostic and Statistical Manual of Mental Disorders, 4th edition symptom criteria for major depressive disorder. The PHQ-9 has been extensively validated in cross-national samples, demonstrating strong evidence of internal consistency, structural validity, and criterion validity against psychological functioning and health care use. Measurement invariance has been demonstrated across racial groups and genders [46-50]. In diverse college student populations, a 1-factor model has been supported [49]. In the current sample, a Cronbach α value of .84 was observed.

#### 7-item Generalized Anxiety Disorder

The 7-item Generalized Anxiety Disorder (GAD-7) is a self-administered, unidimensional instrument for screening Diagnostic and Statistical Manual of Mental Disorders, 4th edition criteria for generalized anxiety disorder. The GAD-7 has demonstrated strong reliability and structural validity evidence among college-attending young adults and, more broadly, evidence of criterion validity against a mental health professional’s diagnosis in the adult population [51-53]. Exploratory and confirmatory factor analyses supported a 1-factor model for the GAD-7 in college student populations [51]. Within this study’s sample, a Cronbach α coefficient of .91 was observed.

#### Suicide Behaviors Questionnaire–Revised

The Suicide Behaviors Questionnaire–Revised (SBQ-R) is a 4-item measure for identifying individuals at risk of engaging in suicidal behaviors. In a diverse mixed clinical and community-based sample, the SBQ-R has demonstrated criterion validity against inpatient psychiatric admission (yes or no), specifically showing high sensitivity (93%) and specificity (95%) among college students [54]. Two previous studies by Osman et al [54] and Aloba et al [55] supported a 1-factor model for the SBQ-R. For the current data, a Cronbach α coefficient of .84 was observed.

### Study Procedure

Each study session involved 2 separate 30-minute components conducted consecutively (up to 48 hours apart). In the first component, a research assistant briefly introduced the HEARTSMAP-U tool and sent participants a secure link to self-administer the web-based tool version, along with 2 additional self-administered psychological instruments: the PedsQL-YA and MHC-SF. The second component, conducted by a research clinician, involved a psychosocial evaluation based on the clinician’s own standards of practice and professional experience. Clinicians were blinded to students’ HEARTSMAP-U assessment results. For each participant, clinicians reported on the presence or absence of psychiatric and broader psychosocial concerns and their respective severity levels and types and appropriate campus and community-based resources. After completing their session, students received a copy of their HEARTSMAP-U report, which included service recommendations triggered based on their HEARTSMAP-U scoring pattern. The same clinician completed a student’s baseline and follow-up assessments to control for interclinician variability. A total of 5 clinicians carried out assessments, 2 mental health nurses and 3 registered counselors, all employed with the UBC Health Service or Counselling Services.

All procedures were repeated at the 3-month follow-up with several modifications. After their HEARTSMAP-U assessment, participants completed a qualitative survey gauging their experiences accessing care following their baseline session. Students were asked to describe whether they had (1) begun accessing care or (2) attempted to access care (unsuccessful) and (3) intended to access care in the future. In addition, students were asked to report any barriers or challenges they experienced in accessing the tool-recommended resources. At follow-up, participants self-reported on a new set of secondary psychological instruments, which included the PHQ-9, GAD-7, and SBQ-R.

### Analytic Approach

#### Predictive Validity

The study was powered to measure HEARTSMAP-U’s sensitivity in detecting psychiatric concerns. On the basis of previous studies, we hypothesized a 2-week psychiatric concern prevalence of 35% and estimated that HEARTSMAP-U would demonstrate 90% sensitivity in identifying psychiatric concerns [41,56]. We considered enrolling 100 students. This sample size would provide ±10% precision with 95% confidence around the 90% estimated sensitivity [57]. We evaluated HEARTSMAP-U’s ability to predict both concern severity and resource needs. First, we calculated the sensitivity and specificity of HEARTSMAP-U in detecting psychiatric concerns (*Psychiatry* domain score ≥1) and assessed its ability to distinguish between mild and moderate or severe issues. Second, we calculated the tool’s ability to identify different psychiatric resource needs: urgent care (eg, crisis line), same-day primary care, nonurgent primary care, counseling services, peer support, and self-directed care. Owing to the small sample size, our resource-related analysis was restricted to the baseline data. All estimates are reported with 95% CIs. We calculated HEARTSMAP-U’s sensitivity and specificity in identifying broader psychosocial issues (eg, housing, relationships, and substance use). The total numbers of true positives, false positives, true negatives, and false negatives are descriptively reported. A summary of our predictive validity analysis has been presented in Multimedia Appendix 3.

#### Convergent Validity

We evaluated the convergent validity between HEARTSMAP-U and comparator instruments using nonparametric Spearman 𝜌 correlation coefficients and 95% CIs. On the basis of Cohen (1988) conventions, absolute correlation values near *r*=0.10 were considered weak, near *r*=0.30 were considered moderate, and near *r*=0.50 were considered strong [58]. All comparisons were decided on a priori. We hypothesized a strong negative correlation (>0.50) between HEARTSMAP-U’s *Psychiatry* domain and both the PedsQL-YA *Emotional Functioning* and MHC-SF *Emotional Well-being* and *Psychological Well-being* subscales. Similarly, we hypothesized a strong negative correlation between HEARTSMAP-U’s *Function* domain and the PedsQL-YA *School Functioning* subscale*.*

Scoring convergence was also evaluated between HEARTSMAP-U’s individual psychiatric sections, *Mood*, *Thoughts & Anxiety*, and *Safety*, and composite scores on the PHQ-9, GAD-7, and SBQ-R instruments, respectively. We hypothesized strong correlations between the total score on the PHQ-9 and the *Mood* section, total score on the GAD-7 and the *Thoughts & Anxiety* section, and total score on the SBQ-R and the *Safety* section.

We used the mutual information method to evaluate the level of agreement among the severity classifications in HEARTSMAP-U’s *Psychiatry* domain, the PedsQL-YA’s *Emotional* subscale, and the total MHC-SF score [59]. Each HEARTSMAP-U domain has 4 severity classifications (no issues, mild, moderate, and severe); the PedsQL-YA has 2 classifications (“not at-risk” and “at-risk” of impaired HRQOL); and the total MHC-SF score produces 3 classifications (“flourishing,” “moderately mentally healthy,” and “languishing”). Agreement on severity classification is crucial because the 2 measures can have high convergent validity but assess different severities, which can impact health decision-making. Multimedia Appendix 3 provides a summary of the hypothesized correlations and classification-related analyses.

#### Clinical Utility

At follow-up, participants completed a survey to measure their experiences accessing tool-recommended resources. HEARTSMAP-U makes two types of support recommendations: (1) self-directed resources for maintaining mental well-being and (2) service-based resources for the identified psychiatric and psychosocial needs. Participants were asked whether they had accessed their respective tool-recommended resources (yes or no). Those who had not were asked whether they intended to access the resources in the future (yes or no) and whether they had tried to access the resources (yes or no). If they had tried, they were asked to describe any barriers they experienced. Those who had not attempted to access the resources were asked to briefly explain why and check off any prespecified barriers, including time, cost, cultural sensitivity, transportation, COVID-19, and service availability. Participants could also add any challenges or issues that they felt were not reflected. We report the proportions of students who had begun accessing the resources, those who tried to access the resources but were unsuccessful, and those who made no attempt to access the resources. We also summarize the barriers, challenges, or explanations for each subsample.

## Results

### Demographic Characteristics

Of the 102 enrolled students who completed informed consent procedures, 100 (98%) students completed all study participation and 2 (2%) dropped out after enrollment but before participation. There was no loss to follow-up between baseline and 3-month follow-up. A total of 530 eligible students expressed interest in the study, the demographic details of whom are summarized in Multimedia Appendix 4. We illustrate participant flow from recruitment to participation in Multimedia Appendix 5.

Among the study participants, balanced distributions were observed for gender, sex, and the year of study. Three-quarters (74/100, 74%) of the participants were enrolled in an undergraduate degree program. A majority were full-time students (97/100, 97%), living off campus (63/100, 63%), single (52/100, 52%), and not currently employed (55/100, 55%). Two-thirds (66/100, 66%) of the participants self-identified with a non-European ethnic background, 70% (70/100) identified as straight, and 20% (20/100) were international students. Similar proportions of students had never (42/100, 42%) or only previously accessed (43/100, 43%) mental health support. A sizable number of students reported living with a learning (12/100, 12%) or physical (6/100, 6%) disability. A complete demographic profile of the study participants is reported in Table 1.

**Table 1:** A descriptive summary of the demographic, lifestyle, and health-related characteristics reported for all the study participants (n=100).

Demographic characteristics			Values
Age (years), mean (SD)			22 (3.4)
**Year of study, n (%)**			
	1	27 (27)	
	2	22 (22)	
	3	31 (31)	
	4	14 (14)	
	≥5	6 (6)	
**Sex, n (%)**			
	Male	48 (48)	
	Female	51 (51)	
	Prefer not to answer	1 (1)	
**Gender identity, n (%)**			
	Man	46 (46)	
	Woman	49 (49)	
	A different gender identity	5 (5)	
**Transgender, n (%)**			
	Yes	0 (0)	
	No	95 (95)	
	Prefer not to answer	5 (5)	
**Sexual identity, n (%)**			
	Straight	70 (70)	
	Gay	8 (8)	
	Bisexual	15 (15)	
	A different sexual identity	4 (4)	
	Prefer not to answer	3 (3)	
**Ethnicity, n (%)**			
	Aboriginal person	5 (5)	
	African	3 (3)	
	East and South Asian	24 (24)	
	European	34 (34)	
	South American	3 (3)	
	South Asian	14 (14)	
	West Asian and Middle Eastern	8 (8)	
	Multiethnic	9 (9)	
Living on campus (yes), n (%)			37 (37)
**Employment status, n (%)**			
	Full time (>30 h/wk)	6 (6)	
	Part time (<30 h/wk)	40 (40)	
	Not employed	55 (55)	
	Prefer not to answer	1 (1)	
**Relationship status, n (%)**			
	Single	52 (52)	
	Dating	37 (37)	
	Common law or married	10 (10)	
	Prefer not to answer	1 (1)	
Full-time student (yes), n (%)			97 (97)
**Program type, n (%)**			
	Undergraduate	74 (74)	
	Graduate	19 (19)	
	Professional	7 (7)	
International student (yes), n (%)			20 (20)
**Accessed any mental health support, n (%)**			
	Currently	29 (29)	
	Previously	43 (43)	
	Never	42 (42)	
**Physical disability, n (%)**			
	Yes	6 (6)	
	No	93 (93)	
	Prefer not to answer	1 (1)	
Learning disability (yes), n (%)			12 (12)

### Scoring Distribution

Most students scored 0 or 1 on the tool sections at both baseline (80/100, 80% to 96/100, 96%) and follow-up (78/100, 78% to 98/100, 98%), with few severe issues reported (1/100, 1% to 5/100, 5%). No significant difference was found in sectional scoring distributions between baseline and follow-up (chi-square test; *P*=.06). Clinicians assessed that most students were not currently experiencing psychiatric issues (49/100, 49% to 57/100, 57%), and the tool scored most cases as “mild” (68/100, 68% to 71/100, 71%). The tool and clinical assessments significantly differed in their classification of psychiatric concern severity (*P*<.001), but within each assessment format, severity classifications remained consistent between baseline and follow-up (*P*=.06). See Tables 2 and 3 for participants’ score distributions on HEARTSMAP-U and psychiatric concerns by severity classification, respectively.

**Table 2:** Students’ score distribution across HEARTSMAP-U’s 10 sections at baseline and follow-up (n=100).

	Tool section	No issue (0), n (%)			Mild (1), n (%)		Moderate (2), n (%)		Severe (3), n (%)			
		B^a^	F^b^	B		F		B		F	B	F
	Housing & Material Security	79 (79)	84 (84)	17 (17)		14 (14)		3 (3)		0 (0)	1 (1)	2 (2)
	Education & Activities	40 (40)	50 (50)	40 (40)		42 (42)		18 (18)		7 (7)	2 (2)	1 (1)
	Relationships	43 (43)	55 (55)	45 (45)		35 (35)		10 (10)		9 (9)	2 (2)	1 (1)
	Thoughts & Anxiety	30 (30)	22 (22)	56 (56)		64 (64)		17 (17)		13 (13)	2 (2)	1 (1)
	Substances & Behavioural Dependencies	40 (40)	42 (42)	55 (55)		56 (56)		5 (5)		2 (2)	0 (0)	0 (0)
	Safety	78 (78)	81 (81)	18 (18)		17 (17)		4 (4)		1 (1)	0 (0)	0 (0)
	Sexual Wellness	78 (78)	82 (82)	9 (9)		6 (6)		10 (10)		8 (8)	3 (3)	4 (4)
	Mood	31 (31)	40 (40)	51 (51)		48 (48)		13 (13)		10 (10)	5 (5)	2 (2)
	Abuse	55 (55)	61 (61)	29 (29)		27 (27)		16 (16)		12 (12)	0 (0)	0 (0)
	Professionals & Resources	59 (59)	73 (73)	25 (25)		19 (19)		11 (11)		7 (7)	5 (5)	1 (1)

^a^B: baseline.

^b^F: follow-up.

**Table 3:** Number of students classified by HEARTSMAP-U and clinicians at each psychiatric concern severity level at baseline and follow-up (n=100).

Assessment type	No issue, n		Mild, n		Moderate, n			Severe, n	
	B^a^	F^b^	B	F	B	F	B		F
HEARTSMAP-U	10	15	68	71	21	14	1		0
Clinician	49	57	28	31	23	12	1		0

^a^B: baseline.

^b^F: follow-up.

### Predictive Validity

At both baseline (100%, 95% CI 93%-100%) and follow-up (95%, 95% CI 84%-99%), HEARTSMAP-U displayed high sensitivity in detecting the presence of any psychiatric concern, as shown in Table 4. Its specificity in distinguishing the presence and absence of psychiatric issues was 25% (95% CI 13%-41%) and 21% (95% CI 11%-34%) at baseline and follow-up, respectively. When the “no issues” and “mild issues” categories were collapsed and treated as a negative screen, the adjusted specificity was high (100%, 95% CI 92%-100%). The initial specificity and high false positive rate all reflected instances where HEARTSMAP-U identified “mild” psychiatric issues, but the clinician identified none. HEARTSMAP-U’s sensitivity in triggering resource recommendations ranged from 33% (95% CI 10%-65%) for counseling services to 89% (95% CI 76%-96%) for self-directed resources (eg, workshops, web-based cognitive behavioral therapy, and self-care apps), as reported in Table 5.

**Table 4:** HEARTSMAP-U’s sensitivity and specificity in predicting any psychiatric concerns (mild to severe) identified through a clinician-administered assessment (gold standard).

			Sensitivity (%; 95% CI)		Specificity (%; 95% CI)		Adjusted specificity (%; 95% CI)		Clinician-identified concerns, n (%)						
									Yes				No		
									TP^a^		FN^b^		FP^c^		TN^d^
**Any concerns**															
	Baseline (n=100)	100 (93-100)		25 (13-41)		100 (0.92-1.00)		51 (51)		0 (0)		37 (37)		12 (12)	
	Follow-up (n=100)	95 (84-99)		21 (11-34)		100 (0.92-1.00)		41 (41)		2 (2)		45 (45)		12 (12)	
**Moderate or severe**															
	Baseline (n=51)	67 (43-85)		N/A^e^		77 (58-90)		16 (16)		8 (8)		6 (6)		21 (21)	
	Follow-up (n=43)	70 (43-95)		N/A		77 (41-83)		7 (7)		3 (3)		8 (8)		25 (25)	

^a^TP: true positive.

^b^FN: false negative.

^c^FP: false positive.

^d^TN: true negative.

^e^N/A: not applicable.

**Table 5:** HEARTSMAP-U’s sensitivity and specificity in predicting the psychiatric support needs identified through a clinician-administered assessment (gold standard) at baseline (n=100).

Support recommendation	Sensitivity (%; 95% CI)	Specificity (%; 95% CI)	Clinician-identified concerns, n (%)				
			Issue			No issue	
			TP^a^	FN^b^	FP^c^		TN^d^
Urgent or severe	100 (3-100)	93 (86-97)	1 (1)	0 (0)	7 (7)		92 (92)
GP^e^ or counseling services	83 (61-95)	40 (29-52)	19 (19)	4 (4)	46 (46)		31 (31)
GP	81 (54-96)	41 (29-52)	13 (13)	3 (3)	50 (50)		34 (34)
Counseling services	33 (10-65)	96 (89-99)	4 (4)	8 (8)	4 (4)		88 (88)
Peer support	88 (62-98)	24 (15-34)	14 (14)	2 (2)	64 (64)		20 (20)
Self-management	89 (76-96)	4 (0-13)	40 (40)	5 (5)	53 (53)		2 (2)

^a^TP: true positive.

^b^FN: false negative.

^c^FP: false positive.

^d^TN: true negative.

^e^GP: general practitioner.

At baseline, HEARTSMAP-U’s sensitivity in detecting the broader psychosocial challenges identified by a clinician-administered assessment ranged from 72% (95% CI 51%-88%) for relationship issues to 100% for substances and behavioral dependencies (95% CI 40%-100%), high-risk sexual behaviors (95% CI 3%-100%), and abuse (95% CI 59%-100%). Specificity ranged from 45% (95% CI 34%-57%) for detecting educational or work-related issues to 86% (95% CI 69%-96%) for detecting housing- and basic need-related issues. As shown in Tables 6 and 7, baseline and follow-up findings were consistent, with the exception of *Housing & Material Security*, where sensitivity decreased to 33% (95% CI 4%-78%).

**Table 6:** HEARTSMAP-U’s sensitivity and specificity in predicting the broader psychosocial concerns identified through a clinician-administered assessment (gold standard) at baseline (n=100).

Section	Sensitivity (%; 95% CI)	Specificity (%; 95% CI)	Clinician-identified concerns, n (%)				
			Yes			No	
			TP^a^	FN^b^	FP^c^		TN^d^
Housing	80 (0.44-0.98)	86 (0.69-0.96)	8 (8)	2 (2)	13 (13)		77 (77)
Education	88 (0.62-0.98)	45 (0.34-0.57)	14 (14)	2 (2)	46 (46)		38 (38)
Relationships	72 (0.51-0.88)	48 (0.36-0.60)	18 (18)	7 (7)	39 (39)		36 (36)
Substances	100 (0.40-1.00)	41 (0.31-0.51)	4 (4)	0 (0)	57 (57)		39 (39)
Sexual	100 (0.03-1.00)	77 (0.67-0.85)	1 (1)	0 (0)	23 (23)		76 (76)
Abuse	100 (0.59-1.00)	65 (0.54-0.74)	7 (7)	0 (0)	33 (33)		60 (60)

^a^TP: true positive.

^b^FN: false negative.

^c^FP: false positive.

^d^TN: true negative.

**Table 7:** HEARTSMAP-U’s sensitivity and specificity in predicting the broader psychosocial concerns identified through a clinician-administered assessment (gold standard) at follow-up (n=100).

Section	Sensitivity (%; 95% CI)	Specificity (%; 95% CI)	Clinician-identified concerns, n (%)			
			Yes		No	
			TP^a^	FN^b^	FP^c^	TN^d^
Housing	33 (0.04-0.78)	85 (0.76-0.92)	2 (2)	4 (4)	14 (14)	80 (80)
Education	75 (0.43-0.95)	53 (0.43-0.64)	9 (9)	3 (3)	41 (41)	47 (47)
Relationships	75 (0.51-0.91)	63 (0.51-0.73)	15 (15)	5 (5)	30 (30)	50 (50)
Substances	100 (0.40-1.00)	45 (0.35-0.55)	4 (4)	0 (0)	55 (55)	43 (43)
Sexual	100 (0.03-1.00)	85 (0.76-0.91)	1 (1)	0 (0)	15 (15)	84 (84)
Abuse	100 (0.40-1.00)	58 (0.48-0.68)	4 (4)	0 (0)	40 (40)	56 (56)

^a^TP: true positive.

^b^FN: false negative.

^c^FP: false positive.

^d^TN: true negative.

### Convergent Validity

As shown in Table 8, strong correlations were found between HEARTSMAP-U’s *Psychiatry* domain and the PedsQL-YA *Emotional* subscale (0.68, 95% CI 0.56-0.78). Similarly, HEARTSMAP-U’s *Psychiatry* domain demonstrated moderate-to-strong correlations with the MHC-SF *Emotional* (0.63, 95% CI 0.49-0.73) and *Psychological* (0.54, 95% CI 0.38-0.67) subscales, as shown in Table 9.

**Table 8:** Spearman rank correlation coefficients^a^ of HEARTSMAP-U domains and PedsQL-YA^b^ subscales.

HEARTSMAP-U	PedsQL-YA, correlation coefficient (95% CI)			
	Emotional	School	Social	Psychosocial summary
Psychiatry	0.68 (0.56-0.78)	0.53 (0.38-0.66)	0.50 (0.34-0.63)	0.69 (0.57-0.78)
Function	0.54 (0.39-0.67)	0.64 (0.50-0.74)	0.46 (0.29-0.60)	0.63 (0.50-0.74)
Social	0.36 (0.18-0.52)	0.33 (0.14-0.49)	0.31 (0.12-0.47)	0.39 (0.21-0.55)
Student Health	0.35 (0.17-0.51)	0.33 (0.14-0.49)	0.34 (0.15-0.50)	0.39 (0.21-0.54)

^a^All correlations are significant at an α of .01 (2 tailed).

^b^PedsQL-YA: Pediatric Quality of Life-Young Adult.

**Table 9:** Spearman rank correlation coefficients^a^ of HEARTSMAP-U domains and MHC-SF^b^ subscales.

HEARTSMAP-U	MHC-SF, correlation coefficient (95% CI)			
	Emotional	Psychological	Social	Total
Psychiatry	0.63 (0.49-0.73)	0.54 (0.38-0.67)	0.53 (0.37-0.66)	0.60 (0.45-0.71)
Function	0.49 (0.33-0.63)	0.55 (0.40-0.67)	0.39 (0.21-0.54)	0.53 (0.38-0.66)
Social	0.31 (0.12-0.48)	0.32 (0.13-0.49)	0.275 (0.08-0.45)	0.34 (0.15-0.50)
Student Health	0.28 (0.09-0.45)	0.35 (0.17-0.51)	0.21 (0.02-0.39)	0.31 (0.12-0.47)

^a^All correlations are significant at an α of .01 (2-tailed).

^b^MHC-SF: Mental Health Continuum-Short Form.

Concern severity classification (none to severe) in HEARTSMAP-U’s *Psychiatry* domain and the PedsQL-YA (“not at-risk” vs “at-risk”) showed significant agreement (Tables 6 and 7). Significant agreement was observed between HEARTSMAP-U’s *Psychiatry* severity classifications and the MHC-SF’s “languishing,” “moderately mentally healthy,” and “flourishing” classifications (Table 10).

For broader psychosocial issues, strong correlation and classification agreement were observed between HEARTSMAP-U’s *Function* domain and the PedsQL-YA *School* subscale (0.64, 95% CI 0.50-0.74). HEARTSMAP-U’s *Mood*, *Anxiety*, and *Safety* sectional scores demonstrated moderate-to-strong correlations with condition-specific instruments: PHQ-9 (0.61, 95% CI 0.48-0.74), GAD-7 (0.71, 95% CI 0.60-0.82), and SBQ-R (0.65, 95% CI 0.60-0.82), respectively.

**Table 10:** Association between severity classifications in HEARTSMAP-U and the PedsQL-YA^a^ and MHC-SF^b^ instruments using measures of mutual information.

First and second instruments			Mutual information agreement, I agreement		Mutual information disagreement, I disagreement		Mutual information, I agreement + I disagreement		Chi-square (*df*)		*P* value		Association outcome^c^
**HEARTSMAP-U Psychiatry**													
	PedsQL-YA emotional	0.25		−0.01		0.24		33.51 (3)		<.001		Agreement	
	MHC-SF total	0.19		0.07		0.26		35.68 (6)		<.001		Agreement	
**HEARTSMAP-U Function**													
	PedsQL-YA work or school	0.18		0.00		0.18		24.99 (3)		<.001		Agreement	
	MHC-SF total	0.10		0.11		0.20		28.12 (6)		<.001		Disagreement	

^a^PedsQL-YA: Pediatric Quality of Life-Young Adult.

^b^MHC-SF: Mental Health Continuum-Short Form.

^c^If I agreement > I disagreement and *P*<.10, then there is a significant agreement [59].

### Clinical Utility

**Figure 2:**
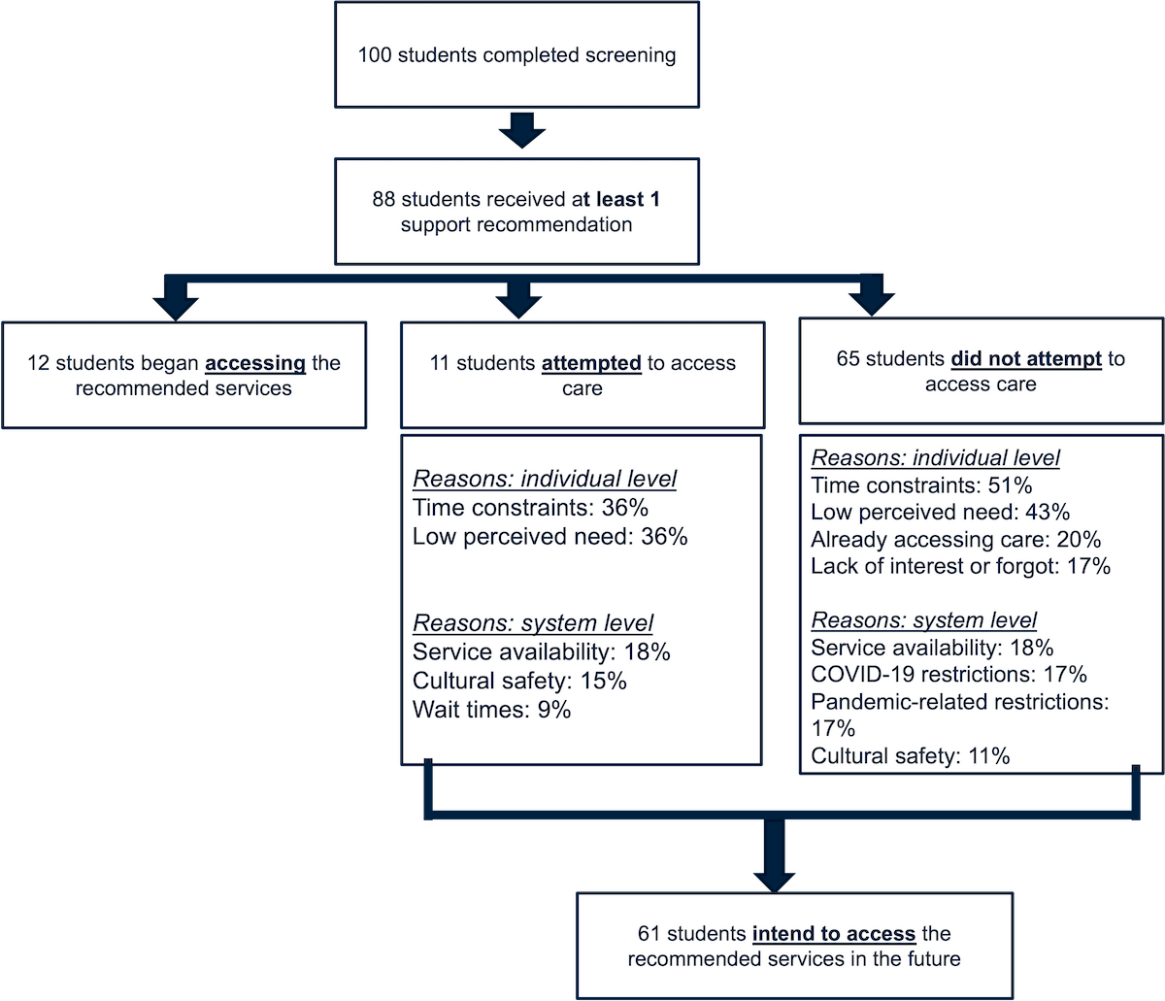
A schematic outlining participants’ connection with psychosocial resources at 3-month follow-up. For a more nuanced understanding of students’ help-seeking, we measured access, attempt, and intention to seek care. As per the British Columbia Centre for Disease Control, culturally safe care is “an outcome based on respectful engagement that recognizes and strives to address power imbalances inherent in the healthcare system. It results in an environment free of racism and discrimination, where people feel safe” [60].

As shown in Figure 2, most participants (88/100, 88%) received at least 1 tool-triggered psychiatric or broader psychosocial support recommendation for the identified needs of any severity level. A smaller fraction (12/100, 12%) of the students solely received recommendations for self-care and maintaining mental well-being. Of the 88 (88%) out of 100 students who received a needs-based support recommendation, 12 (14%) had started accessing the recommended care before their follow-up visit. Accessed services included counseling or primary care services (50/100, 50%), academic advising support (25/100, 25%), and web-based resources (25/100, 25%).

After baseline, 13% (11/88) of the students had attempted to access needs-based services but were unsuccessful in establishing a connection with service providers. Students described the individual- and system-level factors that impeded their service access. At the individual level, time constraints (32/88, 36%) and low perceived need (32/88, 36%) were the most common explanations. At the system level, issues with service availability (16/88, 18%) were the most frequently reported barrier. Additional reasons are outlined in Figure 2.

Of all the students who received needs-based support recommendations (n=88), a total of 65 (74%) students did not attempt to access the specified support. Time constraints (45/88, 51%) and low perceived need (38/88, 43%) were the most common reasons for not accessing care. Additional explanations are summarized in Figure 2. Despite a large proportion of students not accessing care, a majority of these students (54/88, 61%) expressed their intention to do so in the future, either when they perceived the need for them or had more time.

## Discussion

### Principal Findings

The aim of this study was to determine whether HEARTSMAP-U was fit for purpose as a psychosocial self-screening tool for postsecondary students. HEARTSMAP-U displayed high sensitivity and lower specificity in identifying psychiatric concerns and resource needs. Moderate to strong convergent validity evidence was demonstrated between conceptually similar domains in HEARTSMAP-U and other general and condition-specific patient-reported outcome measures. At follow-up, 25% (22/100) of the students who had received service recommendations from HEARTSMAP-U had attempted to access the services. Of those who could not connect with a service provider or those who did not attempt to access care, a majority (61/76, 80%) intended to act on them when they had time or perceived a sufficient need.

HEARTSMAP-U shows high sensitivity in detecting psychiatric and psychosocial concerns and resource needs but consistent low specificity, leading to false positives or overscreening [61]. False positives can be concerning when they result in psychological distress or overwhelm health systems [62,63]. Most of HEARTSMAP-U’s false negatives were for mild support needs (eg, self-direct resources and peer support) or soft recommendations for primary care (“consider accessing...”). To address this, we incorporated an additional recommendation into HEARTSMAP-U’s algorithm, encouraging students experiencing mild issues to secure access to a primary care provider in case of future need escalation (eg, build resource literacy and have a help-seeking plan). Students described soft recommendations as being helpful, “if and when I need it.” Thus, HEARTSMAP-U’s false positives were not perceived by participants as being distressful and are not likely to have significant system-level repercussions. Nevertheless, within the context of standard 2-stage screening procedures [64], HEARTSMAP-U is intended as an initial screening tool and requires follow-up assessment for firm diagnosis and treatment planning.

We observed a moderate-to-high correlation between HEARTSMAP-U’s *Mood* and *Anxiety* sections and total 8-item Patient Health Questionnaire and GAD-7 scores. Consistent with our findings, Alschuler et al [65] observed moderate to high correlation between the College Health Questionnaire and Patient Health Questionnaire (*r=*0.37-0.47), a general screener for common mental health disorders. Similarly, Downs et al [66] showed that the Symptoms and Assets Screening Scale’s *Anxiety Symptoms* and *Depressive Symptoms* subscales were significantly correlated with the Beck Anxiety Inventory (*r=*0.68) and the 8-item Patient Health Questionnaire (*r=*0.73), respectively. HEARTSMAP-U’s convergent validity lends evidence in support of our previously developed conceptual framework. The consistency of these findings with both the College Health Questionnaire and Symptoms and Assets Screening Scale is also promising, as the current work builds on these seminal instruments and extends screening utility beyond assessment but includes resource recommendations and navigational support.

Approximately three-quarters (65/88, 74%) of the participants who received HEARTSMAP-U resource recommendations did not attempt to access care by the 3-month follow-up, with many citing low perceived need and time constraints as common barriers. Similarly, in a random sample of 2785 American college students, Eisenberg et al [67] reported that between 37% and 84% of positive screens had unmet service needs, with a lack of perceived need and a lack of time as the most commonly reported barriers. The extant literature has demonstrated consistently low help seeking and, at best, a preference for informal help seeking (eg, friends and family) among postsecondary students [5,68,69]. Indeed, this may be a concerning observation, as a number of our participants may have benefited from mild-to-moderate mental health resources but did not perceive a need for them, which may result in their concerns escalating and impacting their daily functioning and academic success.

Interestingly, over two-thirds (61/88, 69%) of the students who did not access care expressed an intention to use tool-recommended resources in the future. Although consistent with the existing understanding that students exposed to behavioral persuasion messaging report greater help-seeking intention [70], this study extends this knowledge to the context in which screening interventions are coupled with resource messaging. Students experienced system- and individual-level challenges as barriers to care. Several students (4/100, 4%) called for more self-referral or integrated services, voicing frustrations that even with resource information, accessing care involved too many steps. These comments echo longstanding challenges with screening programs, as their utility is often contingent on system capacity and readiness [17]. In primary care settings, the colocation of behavioral screening with specialists has shown increased referral completion and service use [71]. Similarly, linking HEARTSMAP-U with integrated digital mental health services may reduce logistical barriers (eg, opening new webpages and getting a general practitioner referral) and support more seamless connection with resources after screening. To this effect, we have worked with institutional partners and researchers to embed HEARTSMAP-U within an e-mental health app called “Minder,” which includes built-in and immediately accessible life coaching, e-counseling, peer support, and self-directed resources [72].

This paper describes a comprehensive evaluation of HEARTSMAP-U’s measurement properties and clinical utility. Together, the evidence of HEARTSMAP-U’s validity and students’ intention to seek tool-recommended resources demonstrate that HEARTSMAP-U may be fit for purpose as a psychosocial self-screening tool for the postsecondary educational setting. However, care must be taken to ensure appropriate access to secondary assessments to “rule out” false positives. The National College Health Assessment and Canadian Campus Well-being Survey offer population-level data on student health and academic outcomes [73,74]. However, to our knowledge, these instruments do not offer individual-level information that could facilitate students’ mental health literacy, self-awareness, and understanding of personal support needs. Validated at the individual level, HEARTSMAP-U has the potential to be scaled and offered alongside institution-facing measures to promote measurement that is guided by the principles of student centeredness, as outlined in Canada’s *National Standard for Post-Secondary Student Mental Health and Well-being* [7]*.*

A strength of this study lies in working with campus-based mental health clinicians nurses, as their assessment served as a pragmatic and realistic “gold standard” for the evaluation of predicative validity. This is especially important, as it allowed us to identify nonspecific or subthreshold concerns that may not meet the rigid diagnostic criteria. In addition, the gold standard should be driven by student needs and put their voices first, which is best done by campus mental health clinicians who work with students on a daily basis. Our study was not without limitations. HEARTSMAP-U’s scoring distribution was skewed toward mild to moderate concerns, limiting our ability to validate the instrument’s ability to identify severe psychiatric and psychosocial needs. However, the study’s concern prevalence was consistent with the population-level prevalence and appropriate for the tool’s use as a universal screening measure. Our small sample size also limited the precise evaluation of HEARTSMAP-U’s measurement properties for specific concern severity levels and resource recommendations. Further studies are planned to address these limitations and evaluate HEARTSMAP-U’s predictive performance in different student subpopulations (eg, varying in gender and race).

### Conclusions

Our study offers a preliminary evaluation of HEARTSMAP-U’s measurement properties and clinical utility for multidomain, universal psychosocial screening in the postsecondary educational setting. HEARTSMAP-U displays high sensitivity but lower specificity in identifying psychiatric concerns and resource needs as well as moderate-to-high convergent validity with other conceptually similar instruments. Our results suggest that HEARTSMAP-U has the potential to be scaled and implemented alongside institution-facing measures (eg, early alerts) to promote the prevention and early detection of mental health issues in the learning environment. Studies are currently underway to evaluate the tool’s measurement properties on a larger scale and predictive validity performance across diverse student subpopulations (eg, varying in age, gender, and race).

## Appendix

Multimedia Appendix 1 Variables used to conduct quota-based purposive sampling and target sample sizes for each variable and stratum.

Multimedia Appendix 2 A paper version of the HEARTSMAP-U instrument.

Multimedia Appendix 3 Summary of predictive validity, convergent validity, and severity classification analyses.

Multimedia Appendix 4 Demographic characteristics of the students who expressed interest in participating in the pilot study.

Multimedia Appendix 5 Participant flow and retention from recruitment through study participation.

## Abbreviations

GAD-7: 7-item Generalized Anxiety Disorder
HRQOL: health-related quality of life
MHC-SF: Mental Health Continuum-Short Form
PedsQL-YA: Pediatric Quality of Life-Young Adult
PHQ-9: 9-item Patient Health Questionnaire
SBQ-R: Suicide Behaviors Questionnaire–Revised
UBC: University of British Columbia
