# Supplementary material for: Evaluating the Psychometric Properties and Clinical Utility of a Digital Psychosocial Self-Screening Tool (HEARTSMAP-U) for Postsecondary Students: Prospective Cohort Study
Source: JMIR Ment Health. 2023 Aug 9;10:e48709. doi: 10.2196/48709 (PMC10448294; doi:10.2196/48709)
Supplement: Multimedia Appendix 1 [file mental_v10i1e48709_app1.docx]

| **Variable** | **Strata*** | **N (Total N=100)** |
| --- | --- | --- |
| Gender | Women | 50 |
|  | Men | 45 |
|  | Other gender identities (e.g., non-binary, two spirited) | 5 |
| Program** | Graduate/professional | 25 |
|  | Undergraduate | 75 |
| Racial/ethnic identity | Racialized persons | 45 |
|  | Indigenous persons | 5 |
|  | European descent | 50 |
| Self-perceived mental health status*** | Excellent/Very good/Good | 75-80 |
|  | Fair/Poor | 20-25 |

| *These categories have been aggregated to feasibly determine estimated proportions of students sampled. As shown in our demographic survey many variables will offer additional categories, to understand study population characteristics and generalizability. |
| --- |
| **Based on UBC 2018-2019 institutional demographic data |
| ***These estimates are based on 2019 Canadian Census for ages 18-34 years (general population). Recognizing the ongoing mental health impact of the COVID-19 pandemic, I overestimate ‘Fair/Poor’ status prevalence, compared to pre-COVID estimates in the post-secondary student literature (1,2) |
