# Supplementary figures and images for: Evaluating the Psychometric Properties and Clinical Utility of a Digital Psychosocial Self-Screening Tool (HEARTSMAP-U) for Postsecondary Students: Prospective Cohort Study
Source: JMIR Ment Health. 2023 Aug 9;10:e48709. doi: 10.2196/48709 (PMC10448294; doi:10.2196/48709)

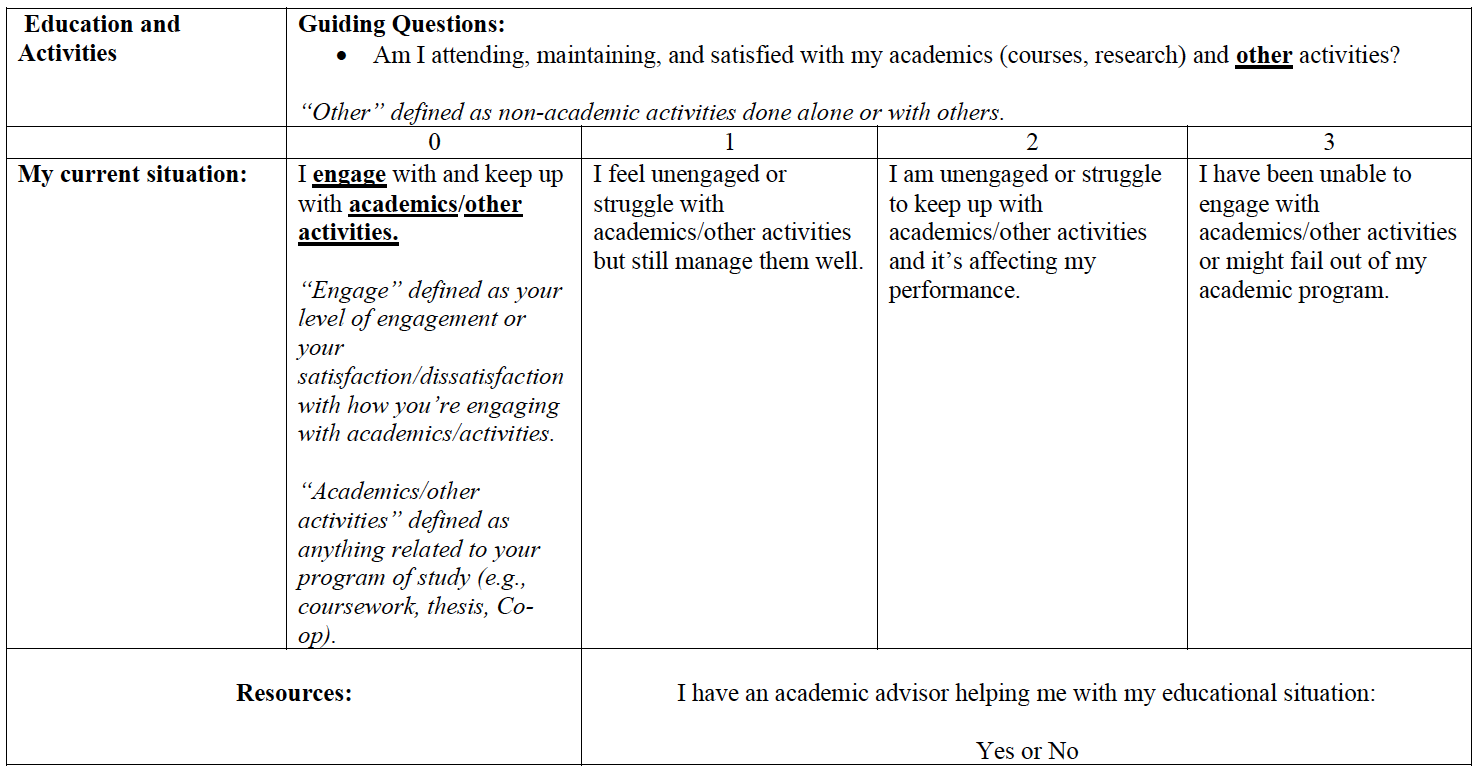

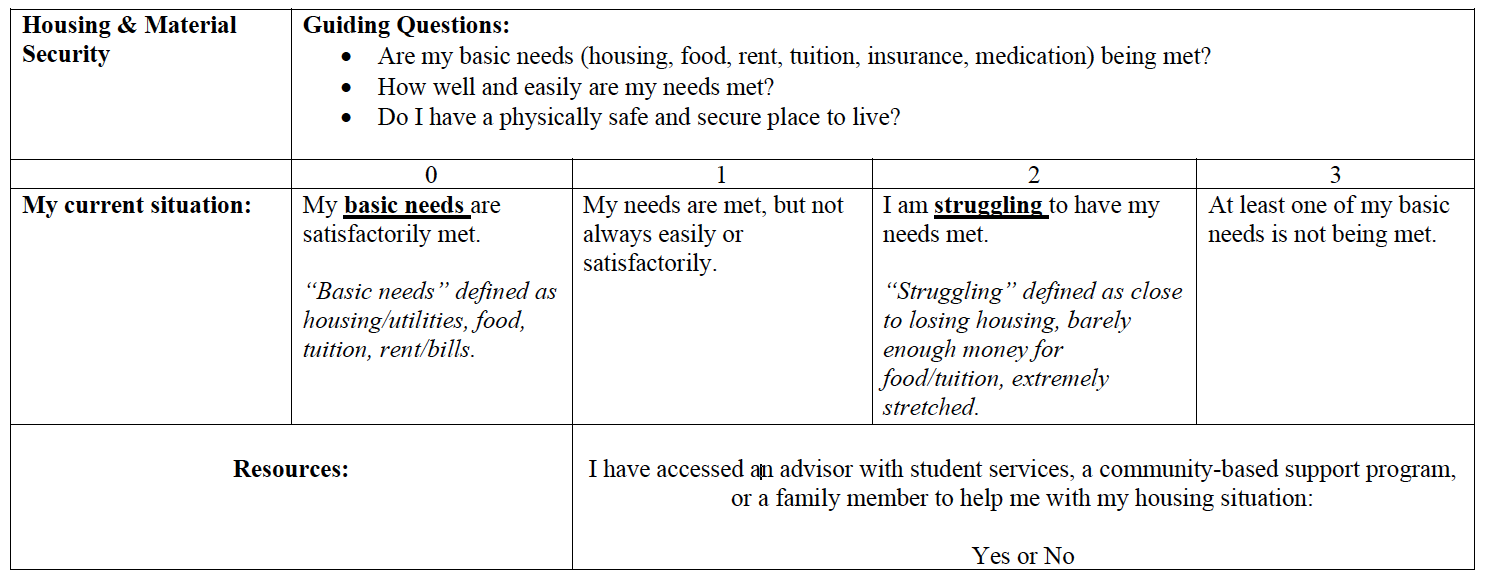


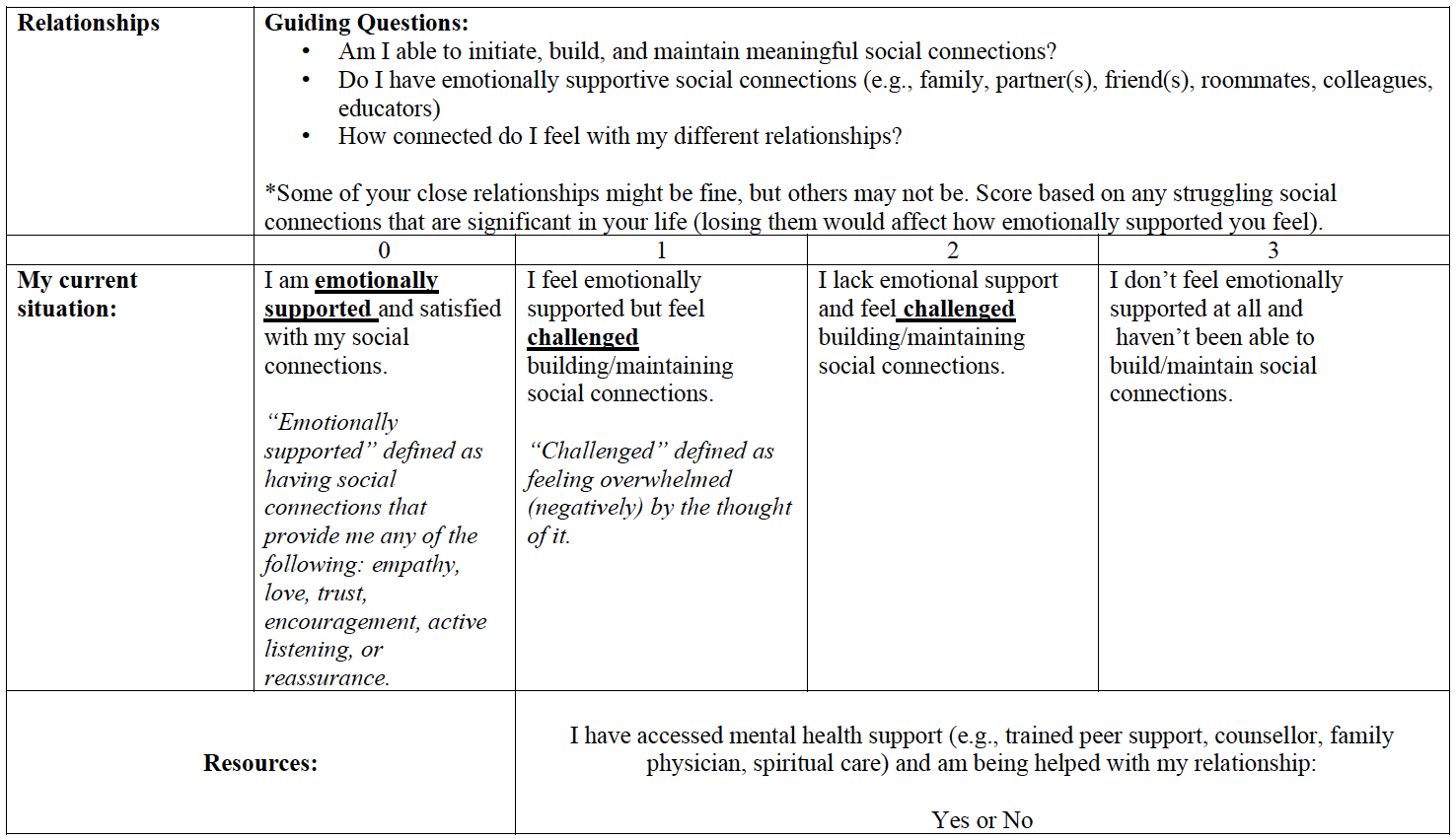


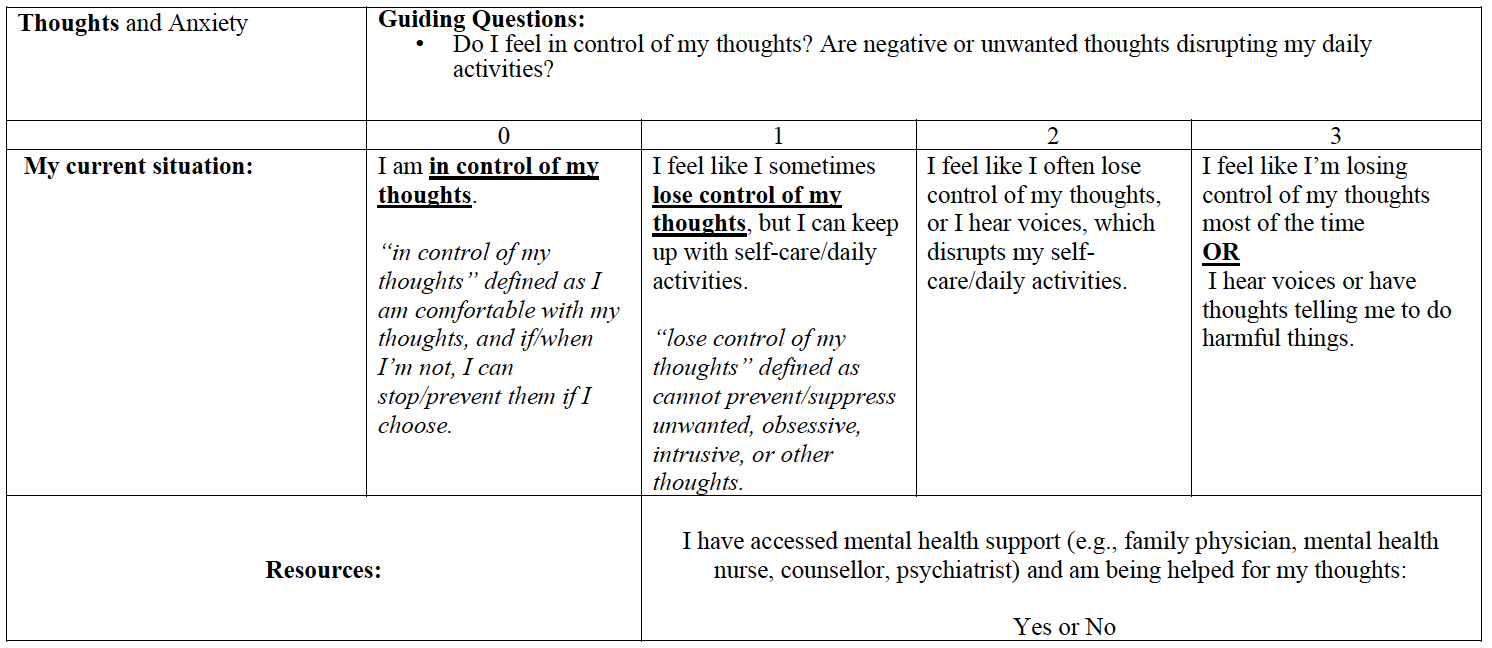


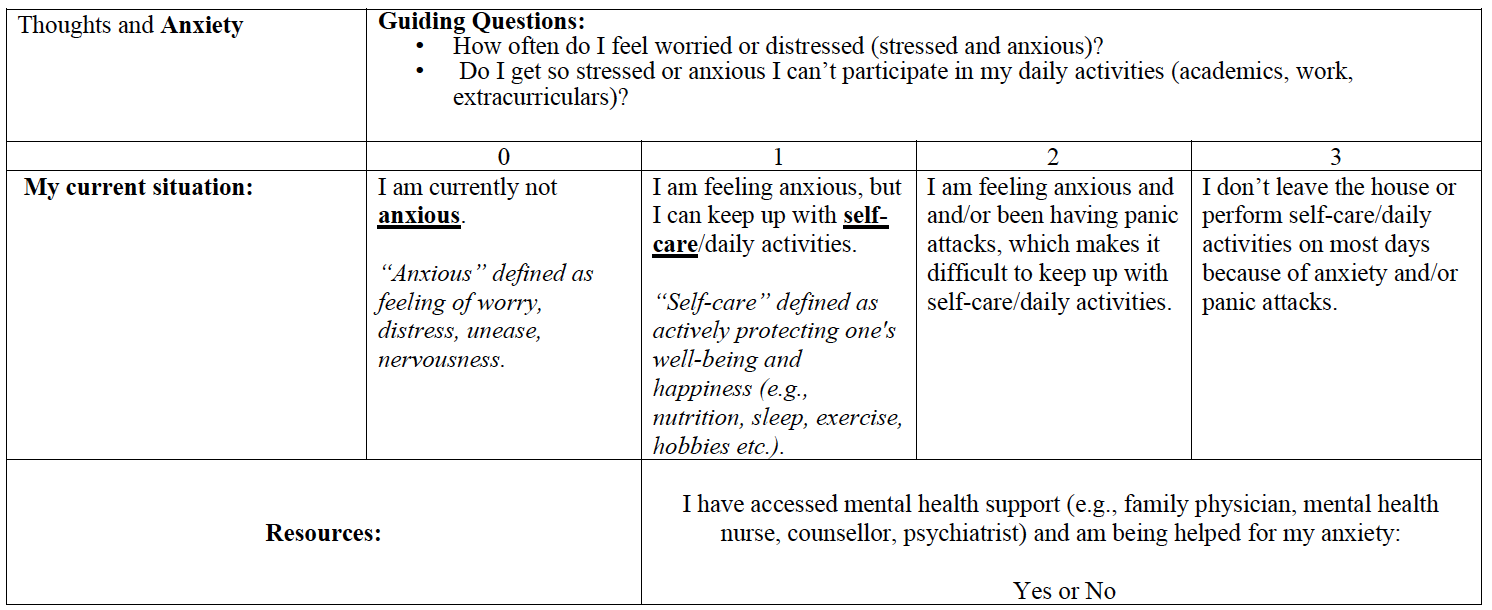


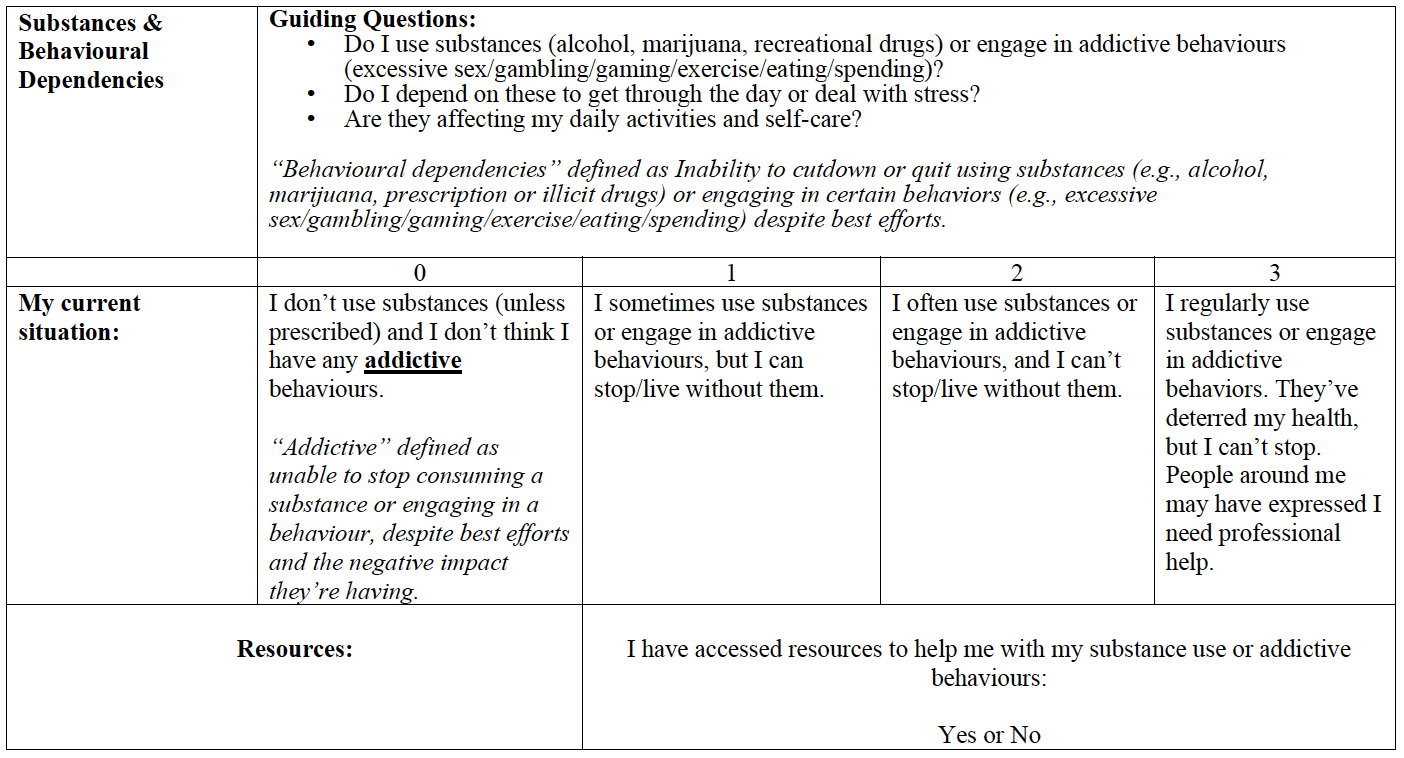


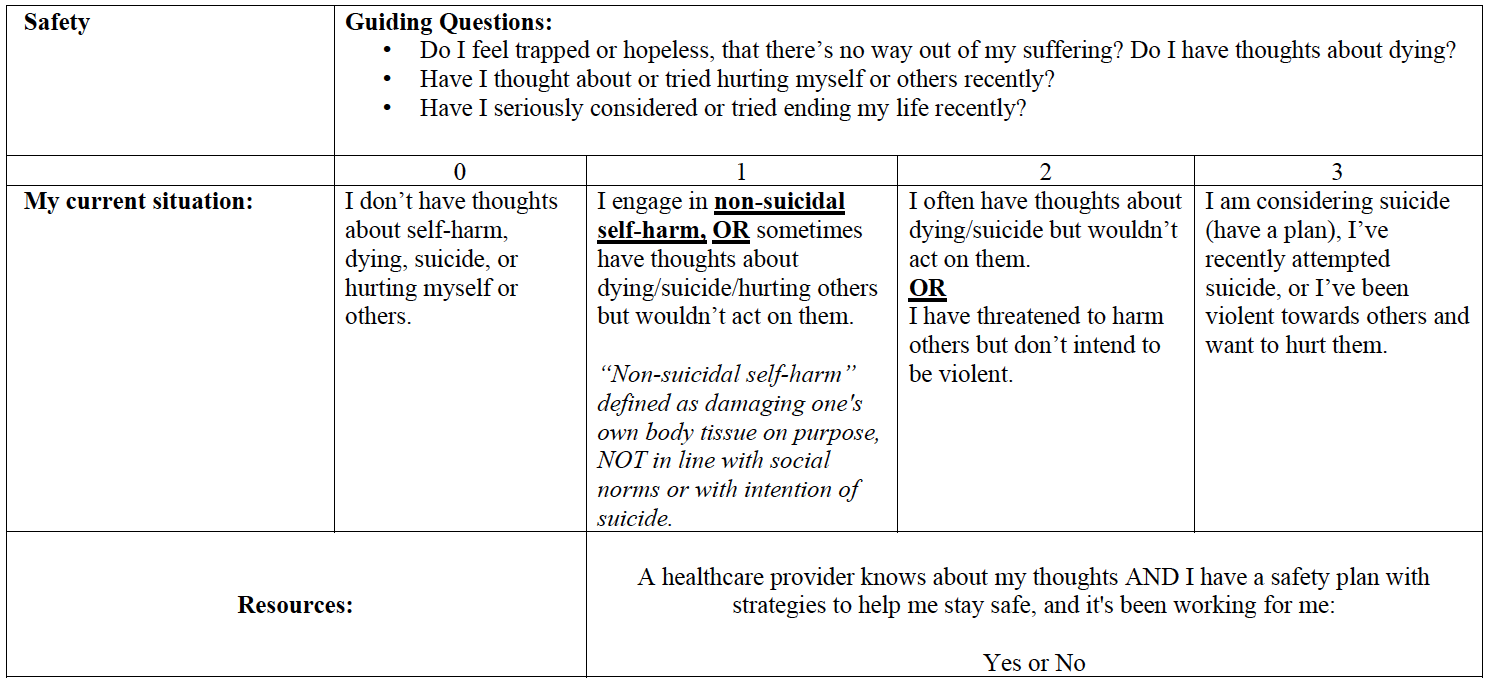


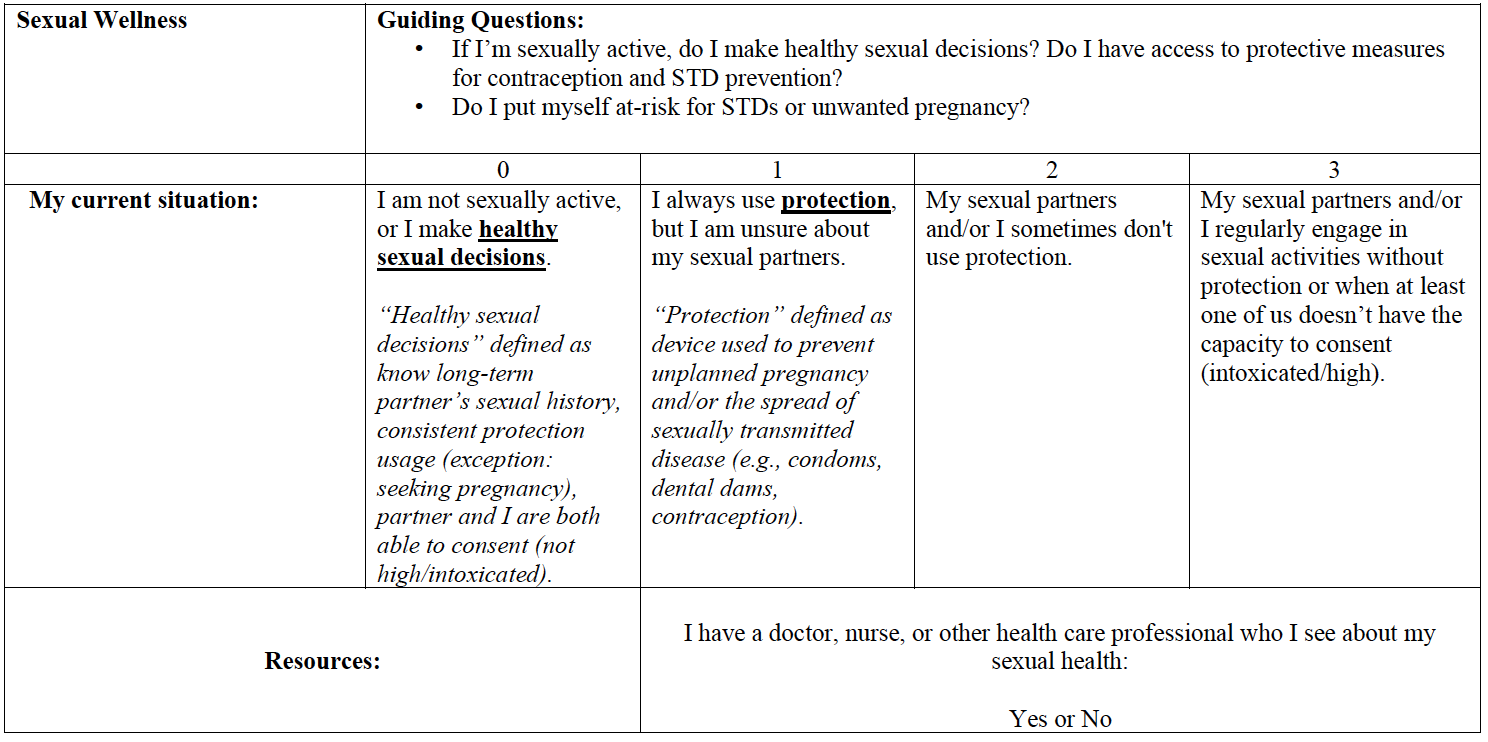


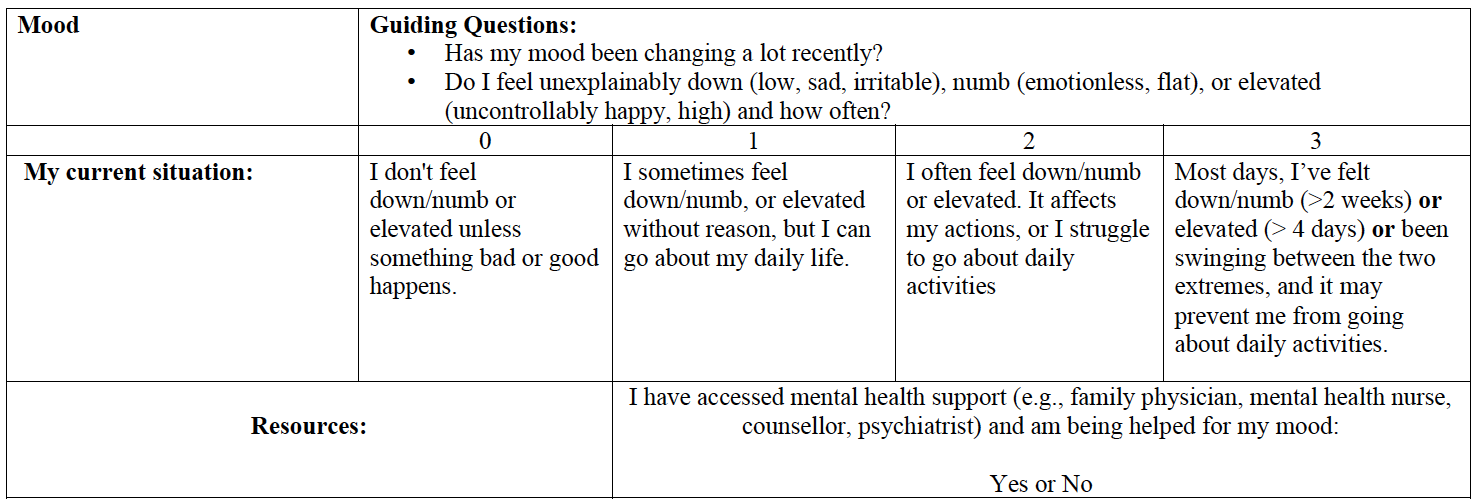


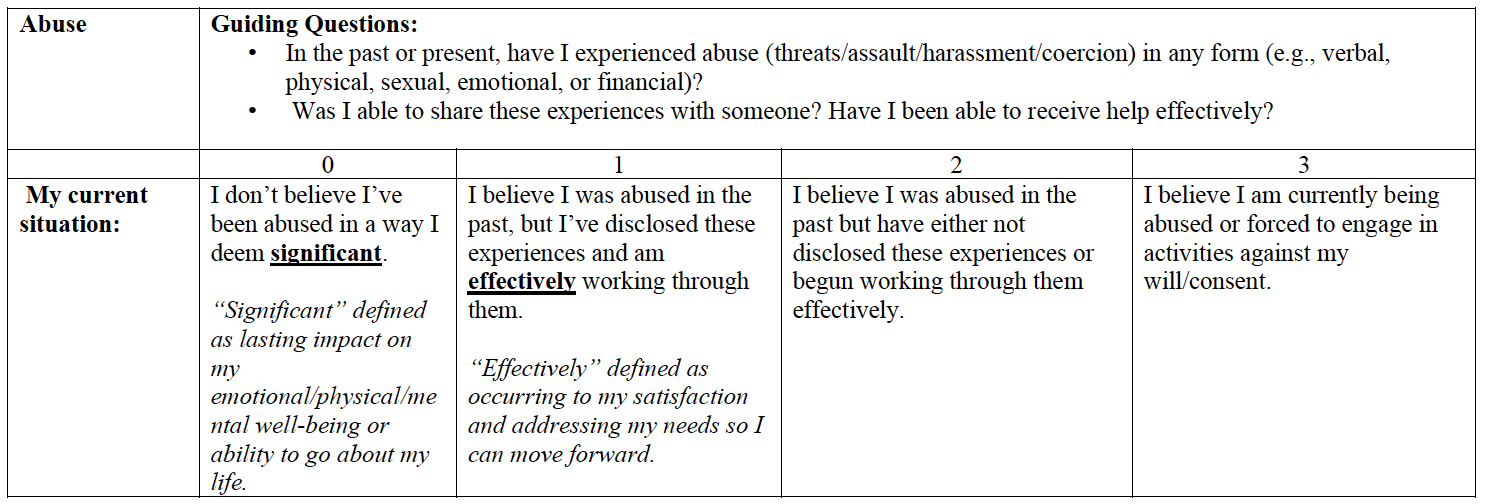

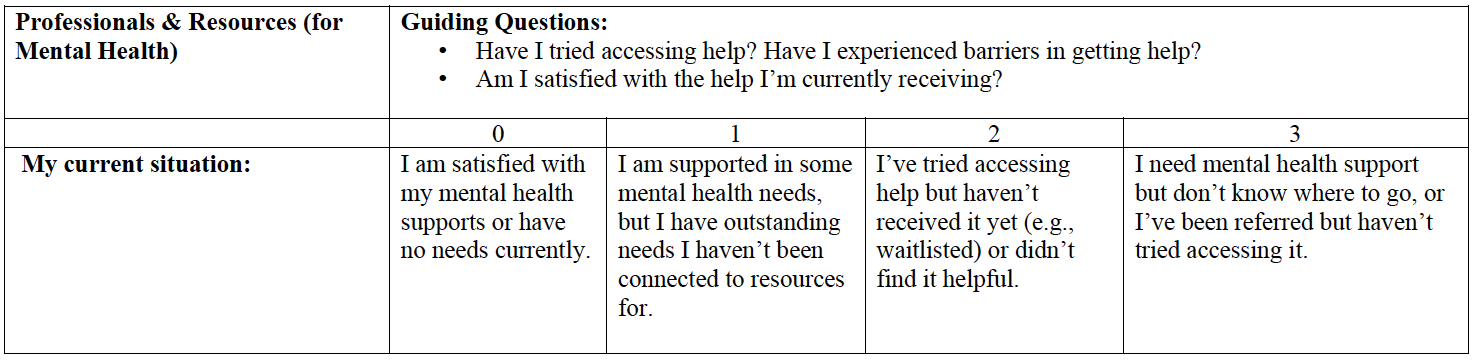

Supplement: Multimedia Appendix 2 [file mental_v10i1e48709_app2.docx]
