# Supplementary material for: Evaluating the Psychometric Properties and Clinical Utility of a Digital Psychosocial Self-Screening Tool (HEARTSMAP-U) for Postsecondary Students: Prospective Cohort Study
Source: JMIR Ment Health. 2023 Aug 9;10:e48709. doi: 10.2196/48709 (PMC10448294; doi:10.2196/48709)
Supplement: Multimedia Appendix 3 [file mental_v10i1e48709_app3.docx]

| **Purpose** | **HEARTSMAP-U** | **Additional Measures** | **Analysis** |
| --- | --- | --- | --- |
| Predictive validity | Any psychiatric concerns, *psychiatry* domain score > 1 (mild to severe) | Clinician-administered interview (criterion): psychiatric concern score > 1 (mild to severe) | Sensitivity, specificity |
|  | Section (per tool section) > 1 | Clinician-administered interview (criterion): each type of psychosocial issue identified (yes/no) |  |
|  | Tool-triggered recommendations | Clinician-recommended services |  |
| Convergent validity | *Psychiatry* domain score | PedsQL-YA *Emotional functioning* score | Correlational analysis |
|  |  | MHC-SF *Emotional well-being* score |  |
|  |  | MHC-SF *Psychological well-being* score |  |
|  | *Function* domain score | PedsQL-YA *School functioning* score |  |
|  | *Mood* score | Total PHQ-9 score |  |
|  | *Thoughts & anxiety* score | Total GAD-7 score |  |
|  | *Safety* score | Total SBQ-R score |  |
| Severity classification | *Psychiatry* domain concern classification: none, mild, moderate, severe | PedsQL-YA *Emotional functioning* classification (at-risk/ not at-risk) | Mutual information analysis |
|  |  | MHC-SF classification (languishing/moderate/flourishing) |  |
|  | *Function* domain concern classification: none, mild, moderate, severe | PedsQL-YA *School functioning* classification (at-risk/ not at-risk) |  |
|  |  | MHC-SF classification (languishing/moderate/flourishing) |  |
