# Supplementary material for: Evaluating the Psychometric Properties and Clinical Utility of a Digital Psychosocial Self-Screening Tool (HEARTSMAP-U) for Postsecondary Students: Prospective Cohort Study
Source: JMIR Ment Health. 2023 Aug 9;10:e48709. doi: 10.2196/48709 (PMC10448294; doi:10.2196/48709)
Supplement: Multimedia Appendix 4 [file mental_v10i1e48709_app4.docx]

| **Demographics** | **N=530** | **%** |
| --- | --- | --- |
| **Gender** | | |
| Male | 152 | 28.7 |
| Female* | 370 | 69.8 |
| Other | 8 | 1.5 |
| **Racial identity** | | |
| White | 143 | 27 |
| Indigenous | 4 | 0.8 |
| Person of Colour | 309 | 58.3 |
| Other | 74 | 14 |
| **Program type** | | |
| Graduate | 146 | 27.5 |
| Undergraduate | 357 | 67.4 |
| Professional | 27 | 5.1 |
| **Perceived Mental Health Status** | | |
| Excellent | 31 | 5.8 |
| Very Good | 116 | 21.9 |
| Good | 192 | 36.2 |
| Fair | 161 | 30.4 |
| Poor | 30 | 5.7 |

* While male and female identifiers were used at the time of this survey, gender typically references to social identities, where terminology such as boys/girls, men/women, or gender diverse peoples, may be more appropriate.
