# Supplementary material for: Evaluating the Psychometric Properties and Clinical Utility of a Digital Psychosocial Self-Screening Tool (HEARTSMAP-U) for Postsecondary Students: Prospective Cohort Study
Source: JMIR Ment Health. 2023 Aug 9;10:e48709. doi: 10.2196/48709 (PMC10448294; doi:10.2196/48709)
Supplement: Supplementary file 5 [file mental_v10i1e48709_app5.docx]

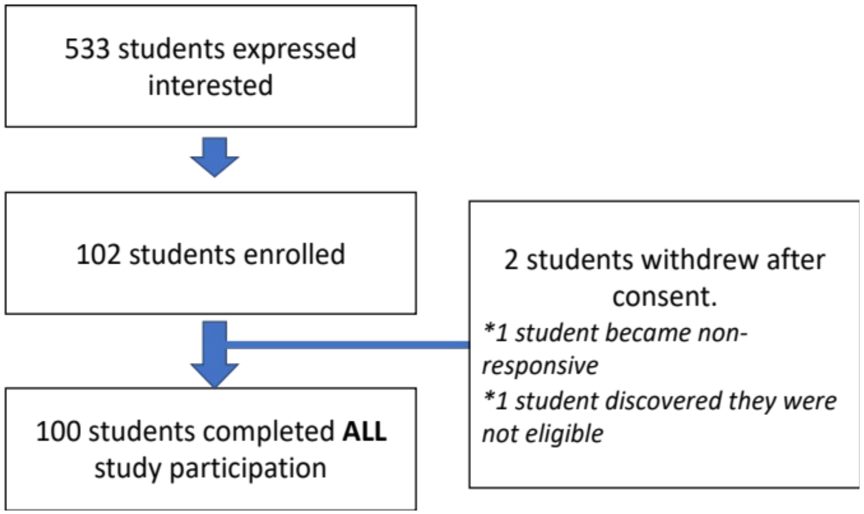
533 students expressed interested
102 students enrolled
2 students withdrew after consent.
*1 student became non-responsive
*1 student discovered they were not eligible
100 students completed ALL study participation
